# Supplementary material for: Nonparametric serial interval estimation with uniform mixtures
Source: PLoS Comput Biol. 2025 Aug 4;21(8):e1013338. doi: 10.1371/journal.pcbi.1013338 (PMC12338840; doi:10.1371/journal.pcbi.1013338)
Supplement: S1 Text — Formulas of performance criteria used in the simulation study and additional simulation results. (PDF) [file pcbi.1013338.s001.pdf]

S1 Text - Supplementary material for: Nonparametric serial interval estimation with uniform mixtures

Oswaldo Gressani[1*], Niel Hens[1,2]

1 Interuniversity Institute for Biostatistics and statistical Bioinformatics (I-BioStat), Data Science Institute, Hasselt University, Hasselt, Belgium
2 Centre for Health Economics Research and Modelling Infectious Diseases, Vaxinfectio, University of Antwerp, Antwerp, Belgium

* Corresponding author. E-mail: oswaldo.gressani@uhasselt.be

Formulas of performance criteria used in the simulation study

Consider M simulation runs and write $\widehat{\theta}_m$ for the estimate of a given serial interval feature θ at iteration m. Also, let $\overline{\widehat{\theta}} = M^{-1}\sum_{m=1}^{M}\widehat{\theta}_m$ be the mean of the estimate in M simulation runs and let $\mathrm{CI}^m_{1-\alpha}(\theta)$ denote the $100(1-\alpha)\%$ confidence interval for θ at iteration m. The bias, empirical standard error (ESE), root mean squared error (RMSE) and coverage probability of 90% ($\mathrm{CP}_{90\%}$) and 95% ($\mathrm{CP}_{95\%}$) confidence intervals are given by:

$$
\begin{aligned}
\mathrm{Bias}(\widehat{\theta}) &:= \overline{\widehat{\theta}} - \theta, \\
\mathrm{ESE}(\widehat{\theta}) &:= \left[\frac{1}{M-1}\sum_{m=1}^{M}\left(\widehat{\theta}_m - \overline{\widehat{\theta}}\right)^2\right]^{1/2}, \\
\mathrm{RMSE}(\widehat{\theta}) &:= \left[\frac{1}{M}\sum_{m=1}^{M}\left(\widehat{\theta}_m - \theta\right)^2\right]^{1/2}, \\
\mathrm{CP}_{90\%} &:= \frac{1}{M}\sum_{m=1}^{M}\mathbb{I}\left(\theta \in \mathrm{CI}^m_{90\%}(\theta)\right), \\
\mathrm{CP}_{95\%} &:= \frac{1}{M}\sum_{m=1}^{M}\mathbb{I}\left(\theta \in \mathrm{CI}^m_{95\%}(\theta)\right).
\end{aligned}
$$

The median width of a $100(1-\alpha)\%$ confidence interval is the median of the differences between the upper bound and lower bound of the constructed confidence intervals in M simulation runs.

Simulation results with three different Gaussian SI target distributions (SARS-CoV-2 Omicron, smallpox and influenza A) and higher average coarseness

**Scenario S1** ($n = 50$)	Bias	ESE	RMSE	$CP_{90\%}$	$CP_{95\%}$	$\Delta CI_{90\%}$	$\Delta CI_{95\%}$
$\mu_{\mathcal{S}}$	-0.016	0.363	0.364	90.10	94.30	1.180	1.400
$\sigma_{\mathcal{S}}$	0.176	0.245	0.301	84.60	91.00	0.743	0.886
$q_{0.05}$	-0.332	0.659	0.738	86.40	91.10	1.916	2.212
$q_{0.25}$	-0.145	0.440	0.463	88.30	93.90	1.417	1.687
$q_{0.50}$	-0.013	0.414	0.414	88.80	94.00	1.307	1.563
$q_{0.75}$	0.113	0.436	0.450	87.80	94.70	1.404	1.680
$q_{0.95}$	0.299	0.655	0.720	84.50	91.20	1.903	2.202
**Scenario S2** ($n = 50$)	Bias	ESE	RMSE	$CP_{90\%}$	$CP_{95\%}$	$\Delta CI_{90\%}$	$\Delta CI_{95\%}$
$\mu_{\mathcal{S}}$	-0.014	0.473	0.473	88.80	94.30	1.540	1.825
$\sigma_{\mathcal{S}}$	0.108	0.333	0.350	86.40	91.80	1.001	1.186
$q_{0.05}$	-0.195	0.902	0.922	84.80	89.10	2.628	2.976
$q_{0.25}$	-0.110	0.586	0.596	87.90	93.50	1.857	2.235
$q_{0.50}$	-0.016	0.547	0.547	88.30	93.40	1.735	2.083
$q_{0.75}$	0.069	0.576	0.580	89.70	94.50	1.855	2.224
$q_{0.95}$	0.194	0.865	0.886	87.00	90.50	2.665	3.000
**Scenario S3** ($n = 50$)	Bias	ESE	RMSE	$CP_{90\%}$	$CP_{95\%}$	$\Delta CI_{90\%}$	$\Delta CI_{95\%}$
$\mu_{\mathcal{S}}$	0.004	0.193	0.193	89.20	94.90	0.620	0.730
$\sigma_{\mathcal{S}}$	0.370	0.124	0.391	5.00	8.40	0.371	0.440
$q_{0.05}$	-0.609	0.327	0.692	40.50	53.70	0.950	1.104
$q_{0.25}$	-0.221	0.214	0.307	70.00	81.00	0.680	0.811
$q_{0.50}$	0.002	0.211	0.211	86.80	93.90	0.646	0.764
$q_{0.75}$	0.230	0.233	0.327	72.90	82.80	0.701	0.828
$q_{0.95}$	0.623	0.306	0.694	46.90	60.10	0.964	1.141

Table S1: Results for Scenarios S1-S3 with $M = 1000$ simulated datasets of size $n = 50$ and censoring distribution $(p_1 = 0.6, p_2 = 0.20, p_3 = 0.10, p_4 = 0.10)$. Scenario S1 assumes a $\mathcal{S} \sim \mathcal{N}(\mu_{\mathcal{S}} = 2.8, \sigma^2_{\mathcal{S}} = 2.5^2)$ target inspired from [1] and imitates the SI distribution of the SARS-CoV-2 Omicron variant. Scenario S2 assumes a $\mathcal{S} \sim \mathcal{N}(\mu_{\mathcal{S}} = 16.7, \sigma^2_{\mathcal{S}} = 3.3^2)$ target inspired from [2] and imitates the SI distribution of smallpox. Scenario S3 is based on a $\mathcal{S} \sim \mathcal{N}(\mu_{\mathcal{S}} = 2.1, \sigma^2_{\mathcal{S}} = 1.2^2)$ target inspired from [2] and imitates the SI distribution of influenza A. The first column contains the selected features of $\mathcal{S}$, namely the mean, standard deviation, 5th, 25th, 50th, 75th and 95th quantiles. Bias, ESE, RMSE, coverage probability (CP) and median confidence interval width (ΔCI) are used as performance criteria.

Simulation results with a Gamma SI target distribution (measles)

**Scenario S4** ($n = 10$)	Bias	ESE	RMSE	$CP_{90\%}$	$CP_{95\%}$	$\Delta CI_{90\%}$	$\Delta CI_{95\%}$
$\mu_{\mathcal{S}}$	0.005	0.768	0.768	84.60	90.90	2.300	2.701
$\sigma_{\mathcal{S}}$	-0.037	0.547	0.548	72.60	78.10	1.308	1.546
$q_{0.05}$	-0.053	1.016	1.017	63.30	66.10	2.000	2.333
$q_{0.25}$	-0.007	0.827	0.827	84.80	88.90	2.580	2.966
$q_{0.50}$	0.056	0.855	0.856	87.20	92.90	2.576	3.192
$q_{0.75}$	0.098	1.027	1.031	85.40	88.40	3.269	3.600
$q_{0.95}$	-0.005	1.763	1.762	59.90	60.60	2.667	3.067
**Scenario S5** ($n = 20$)	Bias	ESE	RMSE	$CP_{90\%}$	$CP_{95\%}$	$\Delta CI_{90\%}$	$\Delta CI_{95\%}$
$\mu_{\mathcal{S}}$	-0.024	0.549	0.549	87.80	93.40	1.725	2.050
$\sigma_{\mathcal{S}}$	0.056	0.394	0.398	82.50	87.40	1.039	1.238
$q_{0.05}$	-0.206	0.731	0.759	79.10	82.20	1.758	2.000
$q_{0.25}$	-0.082	0.585	0.591	88.50	93.50	1.820	2.167
$q_{0.50}$	0.003	0.602	0.602	90.30	94.20	1.921	2.307
$q_{0.75}$	0.084	0.734	0.738	87.40	93.00	2.267	2.740
$q_{0.95}$	0.026	1.212	1.212	78.30	79.10	2.631	3.000
**Scenario S6** ($n = 50$)	Bias	ESE	RMSE	$CP_{90\%}$	$CP_{95\%}$	$\Delta CI_{90\%}$	$\Delta CI_{95\%}$
$\mu_{\mathcal{S}}$	-0.004	0.346	0.346	89.40	94.30	1.120	1.340
$\sigma_{\mathcal{S}}$	0.110	0.262	0.284	86.00	92.60	0.741	0.877
$q_{0.05}$	-0.247	0.473	0.533	83.70	90.10	1.367	1.600
$q_{0.25}$	-0.072	0.373	0.379	89.40	94.60	1.186	1.416
$q_{0.50}$	0.025	0.387	0.387	90.50	95.20	1.243	1.476
$q_{0.75}$	0.095	0.463	0.472	88.70	93.70	1.455	1.749
$q_{0.95}$	0.196	0.812	0.835	86.20	90.10	2.500	2.773
**Scenario S7** ($n = 100$)	Bias	ESE	RMSE	$CP_{90\%}$	$CP_{95\%}$	$\Delta CI_{90\%}$	$\Delta CI_{95\%}$
$\mu_{\mathcal{S}}$	0.014	0.240	0.240	90.00	95.10	0.800	0.955
$\sigma_{\mathcal{S}}$	0.131	0.180	0.223	84.30	91.70	0.558	0.661
$q_{0.05}$	-0.249	0.318	0.404	81.40	88.60	1.003	1.198
$q_{0.25}$	-0.064	0.257	0.265	88.90	94.30	0.855	1.022
$q_{0.50}$	0.042	0.273	0.276	90.00	94.50	0.890	1.058
$q_{0.75}$	0.127	0.321	0.345	89.00	93.50	1.049	1.258
$q_{0.95}$	0.204	0.576	0.610	89.70	93.90	1.767	2.125

Table S2: Results for Scenarios S4-S7 with $M = 1000$ simulated datasets, censoring distribution $(p_1 = 0.8, p_2 = 0.15, p_3 = 0.05)$, $n \in \{10, 20, 50, 100\}$ and target $\mathcal{S} \sim \mathcal{G}(a_{\mathcal{S}} = 17.02, b_{\mathcal{S}} = 1.72)$ with shape $a_{\mathcal{S}}$ and rate $b_{\mathcal{S}}$ inspired from [2] that imitates the SI distribution of measles. The first column contains the selected features of $\mathcal{S}$, namely the mean, standard deviation, 5th, 25th, 50th, 75th and 95th quantiles. Bias, ESE, RMSE, coverage probability (CP) and median confidence interval width (ΔCI) are used as performance criteria.

Simulation results with three different Gaussian SI target distributions (SARS-Cov-2 Omicron, smallpox and influenza A) and $n = 500$ transmission pairs

**Scenario S8** ($n = 500$)	Bias	ESE	RMSE	$\text{CP}_{90\%}$	$\text{CP}_{95\%}$	$\Delta\text{CI}_{90\%}$	$\Delta\text{CI}_{95\%}$
$\mu_{\mathcal{S}}$	-0.006	0.112	0.112	90.50	94.70	0.373	0.444
$\sigma_{\mathcal{S}}$	0.128	0.076	0.149	51.50	64.20	0.254	0.303
$q_{0.05}$	-0.230	0.202	0.306	71.40	82.40	0.679	0.814
$q_{0.25}$	-0.084	0.134	0.158	86.00	92.20	0.454	0.540
$q_{0.50}$	-0.005	0.130	0.130	91.30	95.40	0.425	0.506
$q_{0.75}$	0.088	0.138	0.163	80.90	89.70	0.447	0.536
$q_{0.95}$	0.195	0.216	0.291	80.70	88.30	0.686	0.807
**Scenario S9** ($n = 500$)	Bias	ESE	RMSE	$\text{CP}_{90\%}$	$\text{CP}_{95\%}$	$\Delta\text{CI}_{90\%}$	$\Delta\text{CI}_{95\%}$
$\mu_{\mathcal{S}}$	0.012	0.147	0.147	90.00	96.00	0.489	0.582
$\sigma_{\mathcal{S}}$	0.097	0.103	0.141	78.10	85.90	0.336	0.401
$q_{0.05}$	-0.150	0.273	0.311	84.80	91.80	0.905	1.083
$q_{0.25}$	-0.057	0.175	0.184	89.90	93.80	0.606	0.723
$q_{0.50}$	0.013	0.168	0.168	91.00	95.10	0.565	0.673
$q_{0.75}$	0.074	0.184	0.198	89.40	95.10	0.609	0.725
$q_{0.95}$	0.175	0.301	0.348	84.20	92.70	0.903	1.069
**Scenario S10** ($n = 500$)	Bias	ESE	RMSE	$\text{CP}_{90\%}$	$\text{CP}_{95\%}$	$\Delta\text{CI}_{90\%}$	$\Delta\text{CI}_{95\%}$
$\mu_{\mathcal{S}}$	0.001	0.057	0.057	89.50	94.80	0.190	0.226
$\sigma_{\mathcal{S}}$	0.256	0.038	0.259	0.00	0.00	0.120	0.143
$q_{0.05}$	-0.422	0.124	0.440	0.30	0.60	0.404	0.473
$q_{0.25}$	-0.154	0.059	0.165	13.90	22.80	0.190	0.228
$q_{0.50}$	0.007	0.065	0.065	90.30	94.10	0.212	0.252
$q_{0.75}$	0.151	0.077	0.169	26.00	37.80	0.247	0.290
$q_{0.95}$	0.465	0.096	0.474	1.70	2.50	0.318	0.381

Table S3: Results for Scenarios S8-S10 with $M = 1000$ simulated datasets of size $n = 500$ and censoring distribution ($p_1 = 0.8, p_2 = 0.15, p_3 = 0.05$). Scenario S8 assumes a $\mathcal{S} \sim \mathcal{N}(\mu_{\mathcal{S}} = 2.8, \sigma^2_{\mathcal{S}} = 2.5^2)$ target inspired from [1] and imitates the SI distribution of the SARS-CoV-2 Omicron variant. Scenario S9 assumes a $\mathcal{S} \sim \mathcal{N}(\mu_{\mathcal{S}} = 16.7, \sigma^2_{\mathcal{S}} = 3.3^2)$ target inspired from [2] and imitates the SI distribution of smallpox. Scenario S10 is based on a $\mathcal{S} \sim \mathcal{N}(\mu_{\mathcal{S}} = 2.1, \sigma^2_{\mathcal{S}} = 1.2^2)$ target inspired from [2] and imitates the SI distribution of influenza A. The first column contains the selected features of $\mathcal{S}$, namely the mean, standard deviation, 5th, 25th, 50th, 75th and 95th quantiles. Bias, ESE, RMSE, coverage probability (CP) and median confidence interval width (ΔCI) are used as performance criteria.

References

[1] Kremer C, Braeye T, Proesmans K, André E, Torneri A, Hens N. Serial intervals for SARS-CoV-2 Omicron and Delta variants, Belgium, November 19–December 31, 2021. Emerging Infectious Diseases. 2022;28(8):1699-702.

[2] Vink MA, Bootsma MCJ, Wallinga J. Serial intervals of respiratory infectious diseases: a systematic review and analysis. American Journal of Epidemiology. 2014;180(9):865-75.
