# Supplementary material for: Nonparametric serial interval estimation with uniform mixtures
Source: PLoS Comput Biol. 2025 Aug 4;21(8):e1013338. doi: 10.1371/journal.pcbi.1013338 (PMC12338840; doi:10.1371/journal.pcbi.1013338)
Supplement: Attachment — Submitted filename: AnswersToReviewers_R1.pdf [file pcbi.1013338.s002.pdf]

PCOMPBIOL-D-24-01995 Major revision

We would like to thank all reviewers for their insightful comments as well as the editor for providing additional interesting comments. This review round gave us the opportunity to come up with a much more mature version of the manuscript. In particular, we recently noticed that our nonparametric estimate of the cumulative distribution function can be equivalently reformulated by means of uniform mixtures (a more general formulation). This has important implications for point estimation in the sense that closed form solutions for the estimates of the considered features of the serial interval (mean, standard deviation and quantiles) are available. Furthermore, we reframed our bootstrap sampling scheme in a more traditional way and so it is much clearer how the nonparametric bootstrap resamples the observation set. A direct implication is that our revised nonparametric method is less computationally demanding. We also decided to run entirely new simulation scenarios with only doubly interval-censored serial interval data as double interval censoring should always be adjusted for according to best practices that recently emerged in the infectious disease literature. Finally, our new simulations reveal interesting insights about the proposed nonparametric method, especially regarding the impact of "data coarseness" on estimates. Given that our nonparametric estimate has been reformulated as a uniform mixture model, we changed the title from "Nonparametric serial interval estimation" to "Nonparametric serial interval estimation with uniform mixtures" to make it explicit that the underlying model is a uniform mixture model. Finally, note that the R code to implement our nonparametric method has been removed from the EpiLPS package. This is because Laplacian-P-Splines (LPS) are not used at all in this revised manuscript version and we believe that the proposed nonparametric method (which is not Bayesian and uses the bootstrap) does not really belong to the EpiLPS ecosystem. We therefore decided to include our R code in a new package called EpiDelays. The EpiDelays package is available in a GitHub repository (see html link in our paper).

A detailed point-by-point answer to all the comments is provided below and changes/revisions in the main manuscript are highlighted in blue color for easy tracking and to simplify the review process.

REVIEWER #1:

This manuscript aims to provide a method to estimate the serial interval of an infectious disease using a nonparamtric method. The serial interval is a key parameter to quantify disease transmission, and has important applications to inform disease intervention. First, it is a measure of the epidemic time scale and thus mathematically links the exponential growth rate to the basic reproduction number. This method is commonly used in the early stage of an epidemic, when the exponential growth rate is one of the disease parameters that can be reliably estimated. Secondly, it is commonly used to determine the quarantine period. The key contribution of this paper is the development of an R package that made the proposed method more accessible to epidemiologists. The authors estimated the serial intervals of outbreaks of 2009 influenza A/H1N1 and multiple COVID-19 variants in a range of populations. They should that their estimates are generally inline with parametrized estimations in the literature.

I have a few major concerns:

1. The authors needs to conduct a more thorough the literature review. For example, there are a few systematic reviews of serial interval estimations, especially for the COVID-19 pandemic; see, e.g., Rai et al. (2021) and Alena et al. (2021) below. Interval censored data have also been used in serial interval estimation, albeit using parametric methods; see, e.g., Nishiura (2020) below.

* Rai, B., Shukla, A., & Dwivedi, L. K. (2021). Estimates of serial interval for COVID-19: A systematic review and meta-analysis. Clinical epidemiology and global health, 9, 157-161.

* Alene, M., Yismaw, L., Assemie, M. A., Ketema, D. B., Gietaneh, W., & Birhan, T. Y. (2021). Serial interval and incubation period of COVID-19: a systematic review and meta-analysis. BMC infectious diseases, 21, 1-9.

* Nishiura, H., Linton, N. M., & Akhmetzhanov, A. R. (2020). Serial interval of novel coronavirus (COVID-19) infections. International journal of infectious diseases, 93, 284-286.

We added the above three references in the manuscript.

* Rai, B., Shukla, A., & Dwivedi, L. K. (2021) is cited in the introduction in the following sentence: "*For instance, a systematic review and meta-analysis of serial interval estimates for COVID-19 (Rai, B., Shukla, A., & Dwivedi, L. K., 2021) shows that a majority of studies rely on parametric models with a frequent use of Gamma and Gaussian distributions.*"

* The above reference is also mentioned alongside Alene et al. (2021) in Section 3.1.2, where we describe our new simulation scenarios: "*For each target SI distribution, we simulate $M=1000$ datasets with four different sample sizes $n \in \{10,20,50,100\}$; covering frequently encountered numbers of transmission pairs in the literature (Rai, B., Shukla, A., & Dwivedi, L. K., 2021; Alene et al. 2021); yielding a total of $2\times4=8$ scenarios.*"

* Note that Nishiura, H., Linton, N. M., & Akhmetzhanov, A. R. (2020) was already cited in the introduction in the following sentence: "Parametric methods are by far the most common and usually include the Lognormal, Weibull, Gamma or Gaussian distributions […;Nishiura, H., Linton, N. M., & Akhmetzhanov, A. R., 2020;…]." The latter reference is also used in the applications section, since we use the data provided by Nishiura, H., Linton, N. M., & Akhmetzhanov, A. R. (2020) to illustrate our nonparametric method.

We also added the following relevant references in the introduction section:

* Madewell, Z. J., Yang, Y., Longini Jr, I. M., Halloran, M. E., Vespignani, A., & Dean, N. E. (2023). Rapid review and meta-analysis of serial intervals for SARS-CoV-2 Delta and Omicron variants. *BMC Infectious Diseases*, *23*(1), 429.

* Ward, T., Christie, R., Paton, R. S., Cumming, F., & Overton, C. E. (2022). Transmission dynamics of monkeypox in the United Kingdom: contact tracing study. *BMJ*, *379*.

2. Infection pairs data collected in an early stage of an outbreak may exclude long serial intervals because of the short data collection period, see, e.g., Nishiura et al (2020) above. This may need to be considered in the method.

This is indeed a very insightful comment that unveils an interesting and challenging future research topic around our nonparametric method. In fact, methodological developments aiming at correcting right truncation bias in estimating serial interval distributions have only recently emerged in the literature for parametric settings (see for instance Nishiura et al. 2020, Ward et al. 2022, Park et al. 2024). We believe that extending the latter developments to our nonparametric methodology is outside the scope of this paper which focuses on the adjustment of one of the three biases in delay data outlined in Charniga et al. (2024), namely the double interval censoring adjustment. We added a detailed discussion in our revised manuscript about right truncation (see Section 4), as you rightfully pointed towards the fact that this must be considered (especially in real-time settings during growing epidemic phases where the right truncation problem is sharper as compared to retrospective settings). The added text goes as follows:

"*While our method is specifically tailored for working with serial interval data that has been adjusted for double interval censoring, it is important to highlight that it does not adjust for right truncation. Right truncation means that SI windows are absent from the data because, at the time of data collection, the information required to build a SI window for an infector-infectee pair (i.e. two successive symptom onset times) is not yet available. The problem of right truncation appears in real-time settings and implies an overrepresentation of shorter serial intervals, which in turn can lead to underestimation of SI statistics (Charniga et al. 2024, Nishiura et al. 2020). Right truncation is accentuated during the early stage of an epidemic when it undergoes a growing phase (Park et al. 2024). In retrospective analyses, right truncation is usually not a problem if the surveillance period is long enough to provide a representative sample (Charniga et al. 2024), and in that case, our data-driven approach does not require further adjustment. Methodological developments to correct for right truncation bias in estimating serial interval*

distributions have only recently emerged in parametric settings (Nishiura et al. 2020, Ward et al. 2022, Park et al. 2024). An interesting future research direction would be to extend existing right truncation adjustment approaches to our nonparametric setting."

3. Clinical serial intervals, as stated in the introduction, may be negative, especially for COVID-19 or influenza, because of pre-symptomatic transmissions. The main consequence is that it may not be obvious that the infector is the patient in the pari with the earlier symptom onset date. How do we deal with the uncertainty of who the infector is? This may be further complicated

This is a good and general question that is relevant not only for our proposed nonparametric method, but also any other method attempting to estimate the serial interval distribution (e.g. parametric models where inference is carried out with the likelihood-based or Bayesian approach). Uncertainty about who infected whom is indeed an often-encountered feature in outbreak data and different methods exist to deal with this uncertainty. There are two levels here. The first level encompasses methods that mitigate the risks associated with the uncertainty in transmission links. The second level contains estimation methodologies taking as input the data from the first level that have been corrected for uncertainty in transmission links. Our nonparametric methodology (and actually also likelihood-based/Bayesian methods based on parametric models) act at the second level, and therefore, we believe that it is not worth complicating the manuscript further by providing a technical elaboration on how methods at the first level work. Although the topic of uncertain transmission links is a rather fascinating one, it is not the main topic of this paper. Nevertheless, we provide the following text in Section 2.1 to discuss this:

"To address uncertainty about who infected whom in outbreak data, robust likelihood-based methods can be employed that explicitly account for missing or potentially incorrect transmission links (see e.g. Hens et al. 2012). These methods estimate the probability of transmission between individuals using contact information and the timing of symptom onset. An iterative approach is then used to reconstruct plausible transmission trees and to estimate key epidemiological delay distributions, while simultaneously identifying and mitigating the influence of unreliable data points. An alternative and simplified approach to mitigate the risk of mis-specifying the infector is to concentrate on a subset of transmission pairs for which there is reasonable evidence about who the infector is (Mc Aloon et al. 2021), although this could introduce bias in the analysis."

4. Nonparametric estimation is a useful way to validate parametric estimations, and it avoid the need to assume a distribution a priori. However, many applications requires the distribution itself. For example, the inference of the basic reproduction number from the exponential growth rate requires the calculation of the Laplace transform of the probability density function. For these applications, parametric estimation seems to be easier to work with. Can you explain how your method can be used in such calculations?

Actually, it is also easy to work with our nonparametric estimate of the cumulative distribution function (cdf). As you pointed out, inference of the basic reproduction number via the Lotka-Euler equation requires the calculation of the Laplace transform of the probability density function (pdf). The Laplace transform is usually formulated as a Riemann integral which involves the pdf, but this integral can be easily rewritten in a more general form by using the Riemann-Stieltjes notation which involves the cdf. As such, we can use the Lotka-Euler equation and plug-in our nonparametric estimate of the cdf to obtain an estimate of the basic reproduction number. Of course, the integral has to be solved numerically (which is relatively easy as it is a one-dimensional integral), while for some parametric families, the Laplace transform has a closed form solution. A detailed explanation of this technical point is now given at the end of Section 2.2.

4. This method uses bootstrapping to estimate confidence intervals, which is a commonly used nonparametric method. However, it requires that the errors are i.i.d. yet, the serial intervals may not be i.i.d., because the transmissibility of a patient is typically not a constant. This can be demonstrated from the uncertainties in the figures, where the bands has a width roughly independent of the quantile. This may be quite different from parametric estimations. For example, this may affect the recommendation for quarantine periods.

We recently noticed that our nonparametric estimate can be equivalently reframed as a uniform mixture model (see new Section 2.2) and we have adapted our bootstrap sampling scheme by directly sampling (with replacement) from the observation set since this is faster than our previous approach with the inverse cdf method (see new Section 2.4). Under this new resampling scheme, it appears that the uncertainty bands are not roughly independent of the quantile and tail quantiles tend to have wider widths than, say, the median. As such, we are in alignment with what is usually observed using parametric estimation methods. This is also corroborated by our new simulation settings.

Minor comments:

1. Although the authors give a comparison between their nonparametric estimates with parametric estimates in the literature, it would be interesting to conduct a more thorough comparison of various estimation methods in a table.

We added a comparison of the results for all considered datasets in Table 5.

2. Page 4, line 2, by serial interval times do you mean the symptom onset times?

We meant to describe the coarseness surrounding the (unobserved) serial interval time. We now make a clearer statement of this in the new Section 2.1, where we give a detailed exposition of the coarse structure of serial interval data and also provide a schematic illustration in Figure 1.

REVIEWER #2:

"Nonparametric serial interval estimation" by Gressani and Hens describes a nonparametric approach that applies the inverse CDF method to the empirical CDF of observed serial interval windows. The paper is very clearly written and easy to follow. The results seem promising overall. I think that some aspects of the writing should be expanded upon to clarify the nature of this contribution in light of prior and future work, and particularly to clarify whether the primary focus of this manuscript is Methodological (as I think it is).

Thank you for this positive feedback. We recently noticed that our nonparametric cumulative distribution function estimator can be equivalently reformulated as a uniform mixture model. This has positive consequences regarding point estimates of SI features as the latter have closed form solutions. We also reframed our bootstrap sampling scheme by directly sampling (with replacement) from the observation set since this is faster than our previous approach with the inverse cdf method (see new Section 2.4). To explain this in detail, we entirely rewrote Section 2 with clear subsections that elaborate on the uniform mixture model formulation (Section 2.2), point estimation (Section 2.3) and quantification of uncertainty via the bootstrap (Section 2.4).

Since the authors have published methods to address similar problems in their reference 36 (https://academic.oup.com/aje/advance-article/doi/10.1093/aje/kwae192/7710093) it is a little curious that more direct comparison between the two methods is not made in the present manuscript, especially given their surface-level similarity. It probably behooves the authors to make some more explicit mention on this point.

We added an entire paragraph in the discussion section (Section 4) that elaborates on the main differences between the newly proposed nonparametric approach and our previous work related to incubation times (https://academic.oup.com/aje/advance-article/doi/10.1093/aje/kwae192/7710093). The argument goes as follows:

"There is a surface-level similarity between the nonparametric approach proposed here and our previous work on incubation period estimation (Gressani et al. 2025), however these methods are radically different in several ways. First, in our incubation period paper, we work from a Bayesian perspective and leverage the power and flexibility of Laplacian-P-splines (Gressani et al. 2020; Gressani and Lambert 2018) to estimate the incubation density. The nonparametric approach proposed here is not Bayesian and does not require the specification of a prior. Second, the

distribution of incubation times is modeled in a semiparametric way (by using P-splines) and the model includes spline parameters, while our data-driven method for serial intervals is entirely parameter-free. Third, there is a non-negligible difference in terms of computational complexity. In Gressani et al. (2025), we use Markov chain Monte Carlo (MCMC) to sample from the posterior distribution of the model parameters, while here, the computational cost to obtain estimates of SI features is drastically reduced and only present in the resampling scheme of the bootstrap. Our nonparametric method for serial intervals is also mathematically less technical and thus perhaps more accessible to a broader set of users. For all these reasons, we believe that our approach for estimating incubation times and the newly proposed nonparametric method for estimating the serial interval distribution can be seen as complementary tools."

It also seems to me that the overall focus of this paper is Methodological, rather than conveying new insights from the previously published data. However, the current Applications section (and the total lack of any dedicated subsection for simulation studies) raises the expectation that the paper's findings are primarily epidemiological, when in fact they are not. (As far as I can tell, all of the nonparametric estimates are in agreement with earlier results from parametric methods; this is communicated as an indication that the method is working correctly.)

This is true, the overall focus of this paper is indeed methodological rather than conveying new insights from previously published data. To make this clear, we added a complete section on simulations where we explain the data generating mechanism (Section 3.1.1), provide results of a first set of simulations (Section 3.1.2), explain the negative impact of coarseness (Section 3.1.3) and discuss asymptotic bias (Section 3.1.4). We believe that restructuring the paper that way provides a better alignment with what is expected in PLOS Computational Biology. Also it sheds light on the behavior of our method in different simulation settings.

The main text ought to at least communicate the scenarios where the method succeeds or breaks down. It would be nice if there was a figure summarizing the results of, e.g. the Bias columns from Tables S2-S7 in the main text (or whatever statistic you think is most crucial). I suspect that more than one figure describing the simulation study results in the main text of the paper would greatly assist readers.

The new simulation section (Section 3.1) gives now convincing arguments for when our method succeeds or breaks down (see in particular Section 3.1.3 on the impact of coarseness). Moreover, we provide a figure showing how bias evolves with sample size (Figure 3) which gives a visual overview of the impact of coarseness. We also provide additional simulation results in a supplementary file.

Minor comments:

At the end of the first paragraph in the Introduction, vague reference is made to "best practices" as articulated in [10]. If these can be consolidated into a few points, it would be helpful to readers for these to be described explicitly; or at least, to describe the best practices that your manuscript most closely aligns with

We have identified and enumerated the best practices outlined in Charniga et al. (2024) with which our methodology closely aligns with (see Section 1, Introduction). These are: (1) adjusting for double interval censoring, (2) reporting guidelines for epidemiological delays, (3) accounting for negative serial intervals and (4) reproducibility guidelines.

Also, in the second-to-last sentence of that first paragraph, "transmission interval" is a somewhat confusing or ambiguous term that, if I understand correctly, might just be replaced with the word "quantity"

This sentence has been rephrased.

Third paragraph of Introduction: The explanation for censoring here is nice, although I was not sure what "coarseness" referred to until reaching the end of the paragraph when you mention that symptoms are reported on a timescale of calendar days. For readers trying to work through this introduction on their first pass, it might be helpful if you mentioned the calendar time issue towards the beginning, and then expounded on coarseness in general.

This is a good idea. We now entirely avoid the term "coarseness" in the introduction and wait until Section 2.1 to discuss the inherent coarseness of serial interval data. Note also that we have added a schematic representation (Figure 1) to clearly explain what coarseness means.

Overall comment on the Introduction: I found myself wondering about the role of uncertainty in who-infects-whom. It seems that this method assumes that identities of the individuals involved in the transmission pairs are assumed known without error. I'm mindful that this might be an assumption made in earlier methods using parametric models as well, and don't at all intend to apply an inconsistent standard here, but think that the manuscript would benefit from some discussion on this point. Or, maybe the Conclusion section would be a better place to speculate on how the method might be extended in the future to accommodate errors in the who-infects-whom data.

Uncertainty in who infects whom plays indeed an important role and our approach does not assume that identities of the individuals involved in the transmission pairs are known without error. We argue that there are two levels. The first level is concerned with methods that mitigate the risks associated with the uncertainty in transmission links. The second level contains estimation methodologies taking as input the data from the first level that have been corrected for uncertainty in transmission links. Our nonparametric methodology (and actually also likelihood-based/Bayesian methods based on parametric models) act at the second level, and therefore, we believe that it is not worth complicating the manuscript further by providing a technical elaboration on how methods at the first level work. Although the topic of uncertain transmission links is a rather fascinating one, it is not the main topic of this paper. Nevertheless, we provide the following text in Section 2.1 to discuss this:

"*To address uncertainty about who infected whom in outbreak data, robust likelihood-based methods can be employed that explicitly account for missing or potentially incorrect transmission links (see e.g. Hens et al. 2012). These methods estimate the probability of transmission between individuals using contact information and the timing of symptom onset. An iterative approach is then used to reconstruct plausible transmission trees and to estimate key epidemiological delay distributions, while simultaneously identifying and mitigating the influence of unreliable data points. An alternative and simplified approach to mitigate the risk of mis-specifying the infector is to concentrate on a subset of transmission pairs for which there is reasonable evidence about who the infector is (Mc Aloon et al. 2021), although this could introduce bias in the analysis.*"

Inverse-CDF pseudocode: I confess that line 4 of the pseudocode (Else return s* ...) was not immediately obvious. Could you include a derivation of this, maybe just in the Supplement?

New Section 2.3 on point estimation gives a clearer explanation of how the estimated quantiles are computed.

The bootstrap: is the size of the bootstrap samples, n, the same as the number of observed transmission pairs in D? If so, is there some particular reason for doing so?

Yes, the size of the bootstrap samples is indeed equal to n which is the number of observed transmission pairs. Such a resampling scheme (with replacement) from the observation set is what is usually done in the literature and the idea goes back to Efron (1979) (see also Efron and Tibshirani 1994). There exists another technique known as the 'm out of n' bootstrap where a number m of observations (which is smaller than n) are resampled from the observation set of size n, but the classic bootstrap sampling scheme of Efron (1979) works well in our setting and we believe that there will be no major gain in adopting an 'm out of n' bootstrap scheme here. If you are interested in 'm out of n' bootstrap, we can for instance recommend the following two references:

Lee, S. M. (1999). On a class of m out of n bootstrap confidence intervals. *Journal of the Royal Statistical Society Series B: Statistical Methodology*, *61*(4), 901-911.

Bickel, P. J., & Sakov, A. (2008). On the choice of m in the m out of n bootstrap and confidence bounds for extrema. *Statistica Sinica*, 967-985.

Applications: While these results are all very promising, I could not help but think that organizing these into a table would be a much more efficient way to convey the information, and facilitate rapid comparison between your nonparametric method and earlier parametric methods. The results would also benefit from including some information about the different studies you obtained the data from. For instance, why are the serial interval estimates for H1N1 from New York so different from H1N1 from San Antonio? Did the studies come from schoolchildren of different ages, etc? I understand that it isn't too much work for a reader to examine the references themselves, but your article would be much more self-contained if you elaborated on this a little bit.

We organized our real data results into a table (see Table 5) which facilitates comparison between our nonparametric estimates and earlier parametric estimates. There is indeed a noticeable difference in the datasets for H1N1 New York and H1N1 San Antonio as can be seen from the serial interval windows in Figure 4A and Figure 5A. This paper has a methodological orientation and the objective of the application section is to show that our method can be applied on serial interval data from past outbreaks. We therefore decide not to discuss the background of the studies behind the datasets considered. As you mention, the references provided in our manuscript easily allow the reader to dive deeper into the study designs and data if necessary.

Section 3.3: Illness onset data for 2019-nCoV in Wuhan, China: It seems surprising that the CI95 for the earlier parametric model is so much wider than yours. I would have naively expected estimates from parametric models to be more precise than their counterparts from nonparametric methods. Do you have an explanation for this?

We do not believe that estimates from parametric models are generally more precise compared to estimates obtained from nonparametric methods. This really depends on whether or not the chosen parametric distribution is a good approximating candidate to the underlying true SI distribution.

General comment on figures: I printed these on a black-and-white printer, but the ECDFS are all easily legible.

We now have new figures but they should still be easily legible.

Conclusion: I found this a bit short, and was somewhat disappointed by the rather abrupt end of the paper. Would you care to comment on future extensions to this method that you would hope to implement in the future? Are there obvious next steps that other researchers can take to build on this work? What about elaborating on underlying assumptions of the method (like certainty in the identities of individuals involved in transmission pairs, or population structure)? Is there a distinction to be made between analysis of data collected in outbreak settings vs. endemic settings? What if the serial interval distribution changes over time (due to, say, evolution in the pathogen, implementation of widespread non-pharmaceutical interventions, or changes in age demographics)? Not all of these need to be addressed, but I think elaboration on some aspect of future directions should be included.

The conclusion has entirely been rewritten and is much more complete to avoid an abrupt end of the paper. We now discuss many different aspects related to our nonparametric method, namely: the impact of coarseness, right truncation, differences with respect to our work on incubation times, strengths and limitation, and future research directions.

Supplementary material: Looking through the tables, I was struck by the pattern of CP_90 for q0.95. It looks like Scenario 1 is better than Scenarios 5 or 9, when I would have expected double-interval censoring to produce a worse result than single-interval censoring or no censoring. This (q0.95) is obviously going to be one of the hardest things to estimate, but is there any intuition to convey about why this is happening?

We added two new sections (Section 3.1.3 on the impact of coarseness and Section 3.1.4 on asymptotic bias) to shed light on this. Note that our new simulation settings only account for doubly interval-censored data since double interval censoring is a phenomenon we should always adjust for according to the best practices outlines in Charniga et al. (2024).

REVIEWER #3:

Context

The serial interval (SI) of is defined as the time between symptom(s) onset of the infector and the symptoms onset of the infectee. This time can be negative since there nothing to stop the illness onset time of the infector to be greater than the onset time of the infectee. It is consider as a key quantity in infectious disease modelling since it is often used as a proxy of the generation interval.

When time intervals of illness onset between infectors and infectees are observed, then the most commonly method used to estimate the serial interval distribution is by fitting a parametric distribution (e.g. logNormal, Weibull, Gamma etc) to these data, assuming that they are a random sample from the population. Many of these methods have already been implemented in R packages.

A minimal requirement for SI estimation is to have data on symptom(s) onset times for the infector and infectee. However, serial interval data are often censored; either single, or doubly interval-censored.

This paper

This paper is concerned with estimating the distribution of the serial interval non-parametrically.

The basic idea behind this paper is to (i) view the data from a pair of infector-infectee as two observations from the serial interval distributions, (ii) obtain a continuous estimate of the CDF of the SI distribution by smoothing the empirical CDF using interpolation. One can draw samples from the serial interval distribution using the inverse-CDF method and then use bootstrap to estimate summary statistics of the SI distribution.

After a nice introduction to serial interval estimation, a simple estimator is proposed in Section 2, where in Section 3 application of the proposed method/estimator is done in several publicly availale dataset and compared to parametric alternativess.

Comments

I have very much enjoyed reading this paper; it is very well written, well-structured and easy to read. The proposed estimator is very simple to implement and is already available in an R package (EpiLPS)

We have further restructured the paper for an even easier readability. Note that we removed the routines for serial interval estimation from the EpiLPS package and instead added those routines in another package called EpiDelays. The reason is that in the revised version of our manuscript, the Laplacian-P-Splines methodology which is at the core of EpiLPS is now totally absent in our nonparametric serial interval estimation framework. We thus find it more suitable to add our routines in another package.

My main and only concern about the paper is its level of originality/innovation. It is not entirely clear from Section 2.2 but it appears to be either an application, or a straightforard extension, of the algorithm presented in [38] and [39]. In either case, I feel that this is below the bar for Plos Comp Biology.

Our methodology is in fact building upon existing approaches; for instance as presented in Kaczynski et al. (2012) or also in Bratley et al. (1987). However, our formulation is specifically focusing on the problem of doubly interval-censored data in the setting of serial interval estimation. As such, the estimation framework here is motivated around challenges encountered when modeling epidemiological delays. We argue that this brings a different perspective as compared to what is done in Kaczynski et al. (2012). We do not claim to have invented the piecewise linear cdf as the latter is out there since many years; instead we claim that the piecewise linear cdf appears as an interesting tool

for serial interval estimation and show how to use that tool when working with doubly interval-censored data in an infectious disease modeling setting.

We believe that our revised version has raised the bar in at least three different ways: (1) We have reformulated our nonparametric estimate of the cdf as a uniform mixture model, which is a more general formulation. (2) We discuss the role of coarseness more thoroughly and elaborate on its impact on estimates of serial interval features. (3) We added a complete simulation study that highlights in a clear way how our nonparametric estimates behave.

Furthermore, in addition to the application of the algorithm to real infectious disease outbreak data, it would have been nice to have a simulation-study section to demonstrate the proposed algorithm under different scenarios (e.g. different sample sizes) and comparison against the true serial interval distributions.

Thank you, we added a complete simulation study section (see new Section 3.1) and also further simulation results in a supplementary file.

The font sizes in some figures were pretty small.

We have enlarged the font sizes for all figures in the manuscript.

ADDITIONAL EDITOR COMMENTS :

Thank you for submitting your interesting and well-written manuscript! We apologize for the abnormal length of the review. It was difficult to assemble a panel of reviewers around the New Year, and I appreciate the work done by all reviewers.

The three reviewers were all positive in their general opinion of your manuscript. Two were supportive of a resubmission (with revisions), with one reviewer suggesting that the work did not meet the bar of originality/innovation for PLoS Computational Biology. I can understand both arguments, but I personally consider the method to be new and interesting enough (and of value to the infectious disease and modeling communities) to warrant publication, if a satisfactory resubmission is completed. Therefore, as guest editor, I support a "Major Revision".

I essentially support all of the comments and questions provided by the reviewers and would like to see them addressed in the revision. If you feel a reviewer's question or comment can be addressed in the response letter without requiring adjustment to the text, you are welcome to make the argument for that, but I think most proposed edits (or answers to questions asked) would improve the manuscript and its readability by a broad audience. A few general issues certainly warrant substantial change/additions to the text/presentation:

* Two reviewers wanted to see, within the Introduction, more context of serial interval estimation, with additional citations from the literature. It might be useful to have some of this within the Discussion/Conclusion at the end, where prior methods could be compared to those presented here.

We added citations from the literature that bring more context to the problem of serial interval estimation (see e.g. our response to Reviewer #1). Moreover, we entirely rewrote the Discussion/Conclusion section where we discuss (among others) how our nonparametric methodology differs from our previous work on incubation times.

* There are questions raised about the applicability of the serial interval instrument itself (e.g., infector/infectee discrimination, early epidemic censoring, time variability of the distribution, underlying assumptions) which can be raised in the text and acknowledged/addressed, without any change to the method and results presented.

We addressed the following concerns regarding the applicability of our method. Infector/infectee discrimination → Section 2.1; right truncation → Section 4; and underlying assumptions → Section 2.2.

* There are questions about the bootstrap method, which should be addressed, including the question of i.i.d. random variables and the rationale for obtaining n-element bootstrap samples. Even if answers to these are standard within the technique, it would be useful to remind the readers briefly of the basics of the bootstrap method, and how you are implementing a standard application. I personally would find it useful for you to flesh out a bit more how the samples determine the statistics quoted (mean/median and their confidence intervals). It looks to me like the originally constructed cdf does not directly provide the point estimates, but that these and the intervals are obtained from the bootstrap samples. But, in places the interval bars and point estimates don't seem to line up with the visual distribution of the plotted samples (but this may just be a display issue where density of samples can't be seen). Relatedly, it seems that the bootstrap samples are qualitatively different qualitatively than cdf from original dataset. You obtain the piecewise-linear empirical cdf from the censored data, but then you obtain the bootstrap "samples" as (I assume) step-function cdfs from n samples of the empirical pdf (by inverse-cdf method), which are then smoothed. It's not clear to me exactly how this method precisely fits with bootstrap's "resampling the data" (what is the "data" here, and what are the "samples") and I would appreciate clarification (within the text, if it is useful, or just in your response, if it is obvious!).

Our bootstrap sampling scheme has been reframed (see Section 2.4) and we believe that this brings clarity regarding the rationale for obtaining n-element bootstrap samples and how the bootstrap is used to “resample the data”. This also sheds a brighter light on how the bootstrap samples determine the quoted statistics. The estimated cdf (reformulated in a uniform mixture model) now directly provides point estimates and this is made clear from the closed form solutions provided in Section 2.3.

* The question by Reviewer 1 about how other measures (e.g., basic reproductive number) can be inferred, should be addressed. The idea that "interval estimates of any desired features of S" can be obtained in the method is clear, but some discussion of the next steps would be useful: how can it be used by modelers (or policy-makers) to calculate derived quantities or be used in applications.

This comment has been addressed and we explain how the basic reproductive number can be inferred based on our nonparametric approach in Section 2.2 (see also our response to Reviewer #1).

* As Reviewer 2 notes, the manuscript is essentially a methodological contribution, but the "Applications" section seems to promise novel insights about the analyzed data. Perhaps this section should be something like "Model Verification". Additionally, as multiple reviewers suggested, it would be useful to have at least a summary of the simulation results from the Supplementary Material presented in the main text, as part of "verification". In the comparison with the data (or in the final Discussion/Conclusion section), please make clear the conclusions drawn: is the reader simply to see that your method essentially agrees with prior methods, or do the slightly different results indicate a "better" use of the data than parametric methods? As a methodological contribution, it would be useful (as suggested by Reviewer 2), to have at least a brief discussion of where the method might break down or give results that are at odds with other methods. If it is possible to provide "applications" (without too much additional effort) that give more than a simple verification of the technique, but extend the application to extract new insights from the data, that would certainly be useful to modelers and policy makers and could form a distinct "Applications" section.

Indeed, this manuscript is essentially a methodological contribution and we believe that our revised version makes this point clear. We added an entirely new simulation study section (Section 3.1), where we use a more realistic data generating mechanism that considers the data to be doubly interval-censored following the best practices highlighted in Charniga et al. (2024). Note also that the new simulation section gives insights about when our method might “break down” because of the underlying data coarseness (see also our note on asymptotic bias in Section 3.1.4). In the conclusion, we say that our nonparametric estimate of the cumulative distribution function can be used as a benchmark to visually assess whether a chosen parametric model agrees with our data-driven fit, i.e. as an informal lack-of-fit test. Such a guideline can help users to visually assess whether our nonparametric method makes a

“better” use of the data as compared to a chosen parametric model. As such, it will be useful for modelers and policy makers to extract new insights from the data in future applications.

* It would be useful to have the verification/application data summarized in a table, showing results for all data sets considered.

This is now summarized in Table 5.

-BPH
