# Supplementary material for: Nonparametric serial interval estimation with uniform mixtures
Source: PLoS Comput Biol. 2025 Aug 4;21(8):e1013338. doi: 10.1371/journal.pcbi.1013338 (PMC12338840; doi:10.1371/journal.pcbi.1013338)
Supplement: Attachment — Submitted filename: PCOMPBIOL-D-24-01995-R2.pdf [file pcbi.1013338.s003.pdf]

PCOMPBIOL-D-24-01995 Minor revision

We thank the reviewers and the editor for their time and effort in providing additional comments to our revised manuscript and we appreciate the opportunity to improve it in this second revision stage. All the comments in this "Minor Revision" round have been addressed below on a point-by-point basis and changes in the revised manuscript have been highlighted in blue color for easy tracking.

Additional Editor Comments

Dear Authors,

Thank you for your careful attention to the comments and suggestions by the reviewers and myself on the original manuscript, and for your preparation of an improved revised manuscript. Two of the original reviewers and I have read and commented on your revised manuscript (Reviewer #1 was not available for this re-review). Our suggestions and comments are relatively minor and should be able to be addressed in a "Minor Revision".

As before, I support the comments and suggestions provided by the reviewers, and would appreciate your best efforts to address them and the additional comments I have listed below.

Ben Holder
Guest Editor

* The new interpretation of your cdf through the uniform mixture model is a very nice addition. I would only suggest that the references [45,46] be given slightly more credit in your wording. In the original manuscript, your method was presented as an application of this prior work, but with the new manuscript these are listed simply as related works. I would recommend that your method is presented as an application and extension of that prior work, if that was indeed the origin.

Our cdf estimate is structured around two important concepts. The first is the concept of a mixture model and in particular a uniform mixture model. We have therefore added two additional references that have inspired our mixture specification. In lines 148-149, we write: "*Following previous work on mixtures of uniform distributions (see e.g. Gupta and Miyawaki 1978, Craigmile and Tirrerington 1997), we propose to estimate…*"

* Gupta AK, Miyawaki T. On a uniform mixture model. *Biometrical Journal*. 1978;20(7-8):631-637.

* Craigmile PF, Tirrerington DM. Parameter estimation for finite mixtures of uniform distributions. *Communications in Statistics-Theory and Methods*. 1997;26(8):1981-1995.

The second concept is related to the piecewise-linear structure of the cdf, and following your suggestion, we now give more credit to Bratley et al. (1987) and Kaczynski et al. (2012) by writing (lines 157-159):

"Piecewise-linear cumulative distributions functions are endowed with interesting properties that have for instance been studied in Bratley et al. (1987), Kaczynski et al. (2012). These properties will guide us in computing point estimates of different serial interval features."

As such, it is clear that we apply the properties of piecewise-linear cdfs studied in the latter two references to our serial interval estimation methodology.

* I would eliminate the phrase "interval width" to refer to the window of serial interval widths (line 123), because it could cause confuseion with the width of the serial interval itself. I think "window of serial interval", "window of serial interval width", or just "serial interval window" (used, e.g., in line 145) work well.

To avoid any confusion, we have replaced "interval width" by "SI window width".

* What is the meaning of the "zero" in the inequalities in lines 120-121? Can it be eliminated, or should it appear in Figure 1?

The “zero” in the inequalities in lines 120-121 does not have a particular meaning and we therefore removed it. In fact, the important part e.g. for the infectee is $t_{iL}<t_{iR}$ and it does not matter whether t_{iL} or t_{iR} (or both) are negative in the analysis time domain. What really matters is that the order of days is preserved when mapping calendar dates to analysis times. As such, the “zero” will also not appear in Figure 1.

* Figure 1 could provide a nice graphical visualization of the text in the first paragraph of 2.1, but to me it does not really present enough specific aid to the reader to be useful/included. Figure 1A is just a picture of calendars, but is not related to the timings. The number lines have no indication of the discreteness of days that leads to the coarseness. There is no attempt to present the rule for censoring that is given in lines 110-113. There is no attempt to register Figure 1C with Figure 1B, showing how the censored window is related to the reported values. If specifics like these could be added, without make the presentation too complicated, the figure would be much more useful.

We have improved Figure 1 by adding informative details and without making the presentation too complicated. Figure 1A is now also including color shaded cases representing infector and infectee illness onset timing, respectively. This makes the relation between calendar dates and timings of event explicit. Next, in Figure 1B, we added solid circles to the number lines to indicate discreteness of days and make clear in the title of the panel that these circles stand for the analysis time conversion of calendar dates to integers. Also, to make a link between Figure 1A and Figure 1B, we shaded the circles of the infector and infectee with their respective colors. It is also clear from Figure 1B that the number of steps between the colored circles is equal to the number of steps/days between the calendar dates with illness onset events. The rule for censoring is now made clearly explicit in Figure 1C thanks to the help of the circle representation. Adding “+1” to the left bound of the illness onset time of the infector/infectee generates the required “within a day coarsening”. Note that Figure 1C and Figure 1B are now also naturally related, showing how the censored window is related to the reported values. We believe that the added details make the figure much more useful and give an intuitive graphical representation to explain coarseness.

* Also, in Figure 1D, it seems that two things are being identified: the definition of the serial interval, and its coarseness. Perhaps place the "Serial interval coarseness..." below the number line and title the panel something like "The serial interval and its coarseness"

We followed your recommendation and updated Figure 1D accordingly.

* The set \mathcal{D} (lines 144-145, 182, 196-201) is unclear. It seems to me that you are using two different sets with the same name. One is a set of ordered pairs \mathcal{D} = \left\{ (s_{Li}, s_{Ri}) \right\}_{i=1}^n from which you sample the boostrap set (line 198). And the other is the union of all window endpoints (as defined in line 145), i.e., a simple set of the breakpoints from which you can take "a subset of \mathcal{D}" (line 182). I think this could be made a bit more clear, perhaps just sticking to the first set (the set of pairs), since the union of points really only seems to be referenced at line 182.

We now give a clear definition of the set of observables in Section 2.2 by defining $D_i=(s_{iL},s_{iR})$ as the ordered pair obtained from the SI window of the ith transmission pair and by defining the information set (or set of observables) as $I=\{D_1,\ldots,D_n\}$ for a serial interval dataset involving n transmission pairs. This means that we are now working with a set of ordered pairs as you suggested. As such, the bootstrap sampling scheme is more clearly exposed (see Section 2.4). In fact, a bootstrap sample is simply obtained by sampling (with replacement) n elements (or ordered pairs) from I. This new way of writing entirely avoids the union symbol.

* You seem to be avoiding the explicit introduction and notation for the (uniform) pdf from which the cdf is obtained. It might be useful however to use this, e.g., in line 143: f_{\mathcal{S}_i}(s) \sim \mathcal{U}(s_{iL}, s_{iR}) and then

use this pdf within ordinary integrals over s for the definitions/derivations given in the equations between lines 178 and 179. This notation is more common than the RS integral and could make the presentation more accessible to a broader range of readers. It seems that the RS integral is only presented because notation for a pdf has not been introduced. But I leave it up to you to make this decision.

Our methodology is so to say “cdf-centric” in the sense that the cdf is at the core of our methodology right from the start. Reflecting at the level of the ith transmission pair and adopting Laplace’s principle of insufficient reason, we arrive at a (continuous) uniform distribution of the SI, i.e. Si~U(siL, siR). The pdf and cdf of the latter distribution are sketched below:

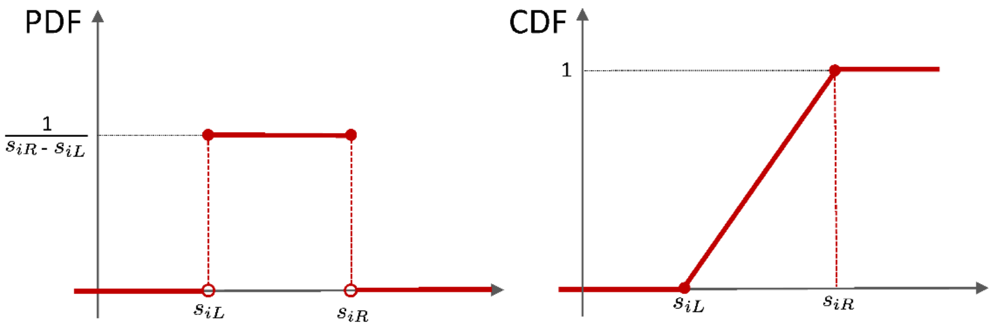

In our model, the serial interval S is a real-valued random variable, i.e. the realization of S can be any real number. The pdf is continuous between siL and siR, however it is not continuous over the entire real line. The cdf however is continuous over the entire real line. When proposing an estimate of the distribution of the SI, it is much easier and also mathematically more convenient to mix a set of functions that are continuous over the real line (i.e. the above cdfs) as compared to mix a set of functions that are plagued with discontinuities. Furthermore, note that for point estimation in Section 2.3, we directly use the cdf to obtain estimates of different quantiles of the SI. This would be more complicated when working with the pdf. Even though the RS integral is less popular than the classic integral with the pdf, we believe that keeping the RS notation in our manuscript is more appropriate because of the central role played by the cdf in our setting.

* In lines 223-227 there seems to be some inconsistency in how you are using \mathcal vs roman font to represent the distribution itself vs. the sample from that distribution.

We have updated our notation in Section 3.1.1 for the sampled uniform random variable. The \mathcal notation is now used for the generated uniform variate so that all \mathcal letters correspond to random variables, except for \mathcal{N} that denotes a Gaussian distribution. We decide to keep the \mathcal{N} notation for the normal distribution as there is no ambiguity that this is indeed a distribution and not a sampled quantity.

* In Figure 3, there is no "A" or "B" (top/bottom?). Also there is no scale on the vertical axis... should there be, or does it not matter?

The letter A and B have been added to Figure 3. There is indeed no scale on the vertical axis. The purpose of this figure is twofold: (1) show how coarseness affects bias versus virtual absence of coarseness and (2) show that we cannot get rid of this bias even with “large” sample size n. Adding the scales on the vertical axis would give us a quantitative idea of that bias as compared to virtual absence of coarseness, but this is not what we want to show here. In fact, bias is already extensively assessed in the simulation settings of Section 3.1.2. Note however that the interested reader can have easy access to this quantitative difference by running the associated R code.

* I agree with the comments of Reviewer 2 about the Discussion. I think it could be also improved by beginning with a short overview paragraph of your results.

The Discussion section (Section 4) has been extended and improved to give a better overview of our results.

* A careful copy editing of the text (particularly new sections/paragraphs) is needed. A few typos appear at lines: 294, 301, 436.

Thank you. We have corrected these typos.

Reviewers' comments:

Reviewer's Responses to Questions

Reviewer #2: The authors have made nice improvements to their manuscript and addressed the concerns I raised. I am glad to recommend acceptance with minor revisions to the writing.

Thank you, the minor revisions are addressed below.

I found the Discussion section a bit terse, overall. I think there might be room to elaborate more on the results about coarseness obscuring the SI distribution, and I think the limitation and future directions paragraphs could be expanded upon.

We have extended the Discussion section and describe our results/method more in detail. We have also added another future direction for our research.

Very minor comments:

Laying out the best practices from [5] toward the end of the Introduction but making reference to those best practices in paragraph 1 reads a little bit disjointed (but thank you for adding the details!)

The reason for citing [5] in paragraph 1 is to make the reader directly aware of the existence of best practices for estimating epidemiological delays. We also wanted to point towards the fact that our methodology aims at being in alignment with such best practices.

Check spelling in 3.1.3: The impact of coarseness - there are several typographic errors

We have corrected the remaining typographic errors.

The tables are a good improvement, but the captions can be totally self-contained to help readers navigate them more rapidly, i.e. should probably define what ESE, RMSE, etc. are within the captions, or at least in Table 1's caption

The performance criteria used in the tables (Bias, ESE, RMSE and coverage/width of CIs) are relatively standard in the literature and we believe that most readers will understand the meaning of these quantities without looking at the formulas. Detailed formulas are included in the supplementary material in case the reader wants more details. In our opinion, adding the latter formulas in the caption of the tables would bring an additional level of reading complexity without being very informative.

Do you need to say something about was assumed about coarsening in 3.2.1 and 3.2.2? It isn't immediately clear to me why you need to "apply" coarsening in 3.2.3 but not the others

Applying coarsening in 3.2.3 is necessary because the original data are simply calendar dates of illness onset for infector-infectee pairs. We now make this clear in Section 3.2.3 by adding: “*Raw data come as calendar dates of illness onset for infector-infectee pairs. We therefore apply a one-day coarsening of the data to recover the desired doubly interval-censored structure.*”

Table 5: I am slightly confused by the apparently inconsistent shading of the rows

Row shading has been removed in Table 5.

Reviewer #3: I would like to thank the authors for taking the time to respond to my questions -- having included a new section with a simulation study and presenting the non parametric estimation as a uniform mixture model has raised the novelty bar and it will be a useful addition to the litereture.
